# Monitoring Protein Fouling on Polymeric Membranes Using Ultrasonic Frequency-Domain Reflectometry

**DOI:** 10.3390/membranes1030195

**Published:** 2011-08-10

**Authors:** Elmira Kujundzic, Alan R. Greenberg, Robin Fong, Mark Hernandez

**Affiliations:** 1 Department of Mechanical Engineering, University of Colorado at Boulder, Membrane Science, Engineering and Technology (MAST) Center, 427 UCB, Boulder, CO 80309–0427, USA; E-Mails: elmira.kujundzic@colorado.edu (E.K.); alan.greenberg@colorado.edu (A.R.G.); 2 Genencor, a Danisco Division, 925 Page Mill Rd, Palo Alto, CA 94304, USA; E-Mail: robin.fong@danisco.com; 3 Department of Civil, Environmental and Architectural Engineering, University of Colorado at Boulder, 428 UCB, Boulder, CO 80309–0428, USA; E-Mail: mark.hernandez@colorado.edu

**Keywords:** proteins, microfiltration, fouling, ultrasonic frequency-domain reflectometry

## Abstract

Novel signal-processing protocols were used to extend the *in situ* sensitivity of ultrasonic frequency-domain reflectometry (UFDR) for real-time monitoring of microfiltration (MF) membrane fouling during protein purification. Different commercial membrane materials, with a nominal pore size of 0.2 μm, were challenged using bovine serum albumin (BSA) and amylase as model proteins. Fouling induced by these proteins was observed in flat-sheet membrane filtration cells operating in a laminar cross-flow regime. The detection of membrane-associated proteins using UFDR was determined by applying rigorous statistical methodology to reflection spectra of ultrasonic signals obtained during membrane fouling. Data suggest that the total power reflected from membrane surfaces changes in response to protein fouling at concentrations as low as 14 μg/cm^2^, and results indicate that ultrasonic spectra can be leveraged to detect and monitor protein fouling on commercial MF membranes.

## 1. Introduction

Fouling is a major problem associated with membrane separation processes because it can often severely limit process performance and selectivity [1]. Fouling typically occurs either on the membrane (external) surface, leading to cake formation, and/or within the internal membrane structure, leading to pore plugging. The efficient microfiltration (MF) of many pharmaceutical products is adversely affected by the microbial cultures and microbiological media biopolymers (*i.e.*, proteins, polysaccharides, and lipids) in the process stream(s) used to produce them. Membrane fouling is a complex phenomenon that depends upon the type of foulant(s), the feed concentration, temperature, pH, and ionic strength, as well as the separation system hydrodynamics. The interplay among these many factors has made a comprehensive understanding of fouling difficult to obtain. There is a significant difference between organic and biological fouling (biofouling). Whereas biofouling is a result of microbial attachment to a membrane and the subsequent growth and release of biopolymers associated with microbial activity, organic fouling on the other hand is often taken to imply the chemical or physical adsorption of organic compounds to the membrane [2]; it follows that organic fouling accompanied biofouling.

It is the primary amino acid sequence that dictates the three-dimensional conformation and surface-charge distribution of proteins; this in turn drives their removal by size exclusion, their sorption potential for partitioning into/onto membranes, and their ability to be denatured and removed using physical and chemical methods. Proteins are divided into crudely defined classes given their size, water solubility, tertiary structure and activity (*i.e.*, enzymes), and no unified system exists for characterizing the thousands of polypeptide sequences that have been catalogued within proteomic databases. Because of their unique and complicated biochemistry, only a few purified proteins have been used as surrogates to model the fouling behavior of accepted protein classes during conventional membrane-challenge studies including water-soluble bovine serum albumin (BSA), some common enzymes (e.g., lysozyme), and poorly soluble structural proteins (e.g., collagen) [3,4,5]. The causes and effects of protein fouling are numerous and have been extensively reviewed and studied [6,7,8].

Persson and colleagues [9] studied and reported the effect of pH on BSA transmission through two different MF membranes. Findings of this study showed that transmission of BSA was highest for a low-protein binding membrane but that protein-associated fouling was significantly affected by feed pH. At pH 5, which is close to the isoelectric point of BSA, the transmission was near 100%; at pH 3 or 7, BSA transmission was significantly lower, while it was dramatically increased when the ionic strength of the BSA solution was increased at pH 3 or 7.

Toussaint and colleagues [10] studied the influence of temperature on the recovery of extracellular *α*-agarase enzymes from fermentation broth using a polypropylene hollow-fiber filter with 0.5 μm pores. Whereas results at 37 °C evidenced higher permeate flux as compared to 22 °C, the study suggested the latter temperature would lead to greater overall enzyme recovery due to better molecular stability at lower temperatures. Kelly and Zydney [11,12] studied mechanical aspects of protein fouling on MF membranes and isolated two distinct mechanisms: deposition of large protein aggregates (size exclusion), as well as sorption and chemical attachment of proteins to other surface-associated deposits.

Characterization of protein-fouled membranes is crucial to understand fouling mechanisms as well as to minimize fouling maintenance and membrane cleaning cycles [13]. A promising approach for improving the understanding and control of spatially defined fouling mechanisms involves the application of practical models and non-invasive, real-time monitoring, which can be validated using data from actual membrane operations. Clearly, there are significant benefits in employing non-destructive methods [14] that are sensitive only to changes in mass accumulating on a membrane surface or to material that “fills” membrane pores, where these markedly different fouling mechanisms can be isolated from each other. A practical methodology that currently satisfies these criteria is ultrasonic reflectometry (UR). UR shows promise in distinguishing among different fouling modes, including internal pore blockage and surface-cake build-up. Recent reports have described the ability of UR to monitor the development of fouling layers on the surfaces of flat sheet [15,16,17,18,19,20,21,22,23] and hollow-fiber membranes [24,25] used for drinking water treatment. Ultrasonic time-domain reflectometry (UTDR) has also been successfully used to detect protein fouling on tubular ultrafiltration (UF) membranes [26]. In the study by Li and colleagues [27], UTDR was used to detect protein on polysulfone (PS) UF membranes fouled with 0.5 g/L BSA solution. Results showed good correspondence between ultrasonic signal responses and the development of BSA association within the membrane microstructure. This study also showed that surface-associated BSA deposits were thicker at neutral pH than at its isoelectric point and that gel layers deposited were more compressible near the isoelectric point than at neutral pH. Results also suggested that BSA partitioning behavior in some membranes varied significantly in response to pH; at neutral pH, protein BSA deposits predominantly on a membrane surface; however, around its isoelectric point, BSA readily sorbs both in and on membranes.

We report here how ultrasonic frequency-domain reflectometry (UFDR) was used to detect and monitor protein fouling on commercial polymeric MF membranes using novel data analysis of frequency shifts in conjunction with wave attenuation. The bulk density of organic fouling layers developing on MF membranes is often only 0.5% different than that of water, making the process of fouling characterization using optical, ultrasonic or piezoelectric contrast from water-based media less sensitive; thus, conventional means of identifying energy “echoes” using waveform amplitude changes from time-domain data are not typically definitive. The type of analysis described herein provides local information regarding morphological changes on membrane surfaces on a three-dimensional microscale which can provide topographic quality and spatial mapping ability.

The objectives of this study were the following: (1) explore the ability of UFDR to detect and monitor protein fouling associated with membrane surfaces in flat-sheet cells operating in a laminar cross-flow regime; and (2) explore the use of UFDR to monitor protein fouling in which membrane types, proteins, and protein concentrations are varied. Using conventional flow-based measurements with a suite of optical, ultrasonic and gravimetric bioassays, we describe studies in which two different commercial MF membranes were systematically challenged with proteins at selected concentrations and discuss leveraging UFDR methodology to monitor and map membrane protein fouling.

## 2. Materials and Methods

### 2.1. Membranes Preparation

Two different commercial polymeric membranes, polyvinylidene fluoride (PVDF) with a nominal pore size of 0.22 μm and polysulfone (PS), with a nominal pore size of 0.2 μm, were used in the experiments. The PVDF membrane is approximately 125 μm thick and is not supported by a separate layer. Our displacement porometry results for this membrane showed a mean pore diameter of 0.20 ± 0.02 μm that agreed well with literature-reported data [28]. The PS membrane is approximately 80 μm thick and was used with a support layer that is ∼200 μm thick. We analyzed this membrane using displacement porometry, and results indicated that the mean pore diameter of the membrane was 0.23 ± 0.03 μm. New membrane coupons (20 cm × 12 cm) were cut from the same membrane roll and prepared according to manufacturer recommendations. Residual preservative agents were removed from the PVDF membrane surface by soaking membrane sample pieces in 70% isopropyl alcohol for 10 min, followed by 2 h soaking in ultrapure water to ensure that the pores were free from residual alcohol. Polysulfone membrane sample pieces were soaked for 30 min in an alkaline solution (ultrapure water, pH adjusted to 10.5–11.0 using 1 M sodium hydroxide) that was constantly mixed at 50–55 °C. After soaking, the membrane was rinsed with ultrapure water to ensure that the pores were free from residual caustic.

### 2.2. Cross-Flow Cell System Integrated with Ultrasonic Instrumentation

**Figure 1:**
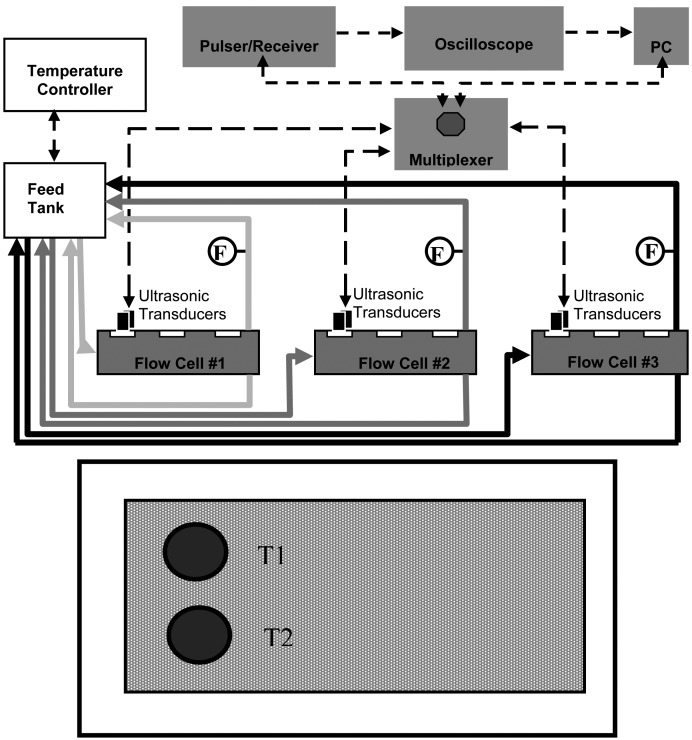
TOP: schematic of microfiltration (MF) cross-flow cell system with integrated instrumentation; BOTTOM: Top view of the bottom plate of the cross-flow cell showing the location of the two ultrasonic transducers (T1 and T2) and flow-sensors (F).

For this study three identical acrylic cross-flow membrane filtration cells were operated in parallel. A schematic of the MF cross-flow cell system with integrated instrumentation is shown in Figure 1 (top).

The dimensions of the cross-flow cell were 4 mm (height) by 150 mm (length) and 102 mm (width); a double “O-ring” arrangement provided a leak-proof seal at the required pressures. The membrane (permeation area *ca*. 150 cm^2^) was supported by a 2-mm thick stainless steel plate. The feed suspension was circulated through the system by three high-pressure pumps (model GBP35.PVSA pump with a model DC305A motor, Micropump, Vancouver, WA, USA); the circulation flow-rate was controlled via a voltage output to the pump motor from a DC-regulated power supply (model 1688A, BK Precision, Yorba Linda, CA, USA).

Two 10-MHz planar ultrasonic transducers in a 1.27-cm diameter element (model V111, Panametrics, Waltham, MA, USA) were mounted on each cross-flow cell for continuous monitoring. Ultrasonic transducers were positioned 2.5 cm from the flow cell feed intake such that the transducers were approximately 3 cm from each other (see Figure 1 bottom). An ultrasonic pulser/receiver (model 505PRX, Panametrics, Waltham, MA, USA) in combination with the ultrasonic transducers and a digital storage oscilloscope (model TDS3052, Tektronix, Richardson, TX, USA) were used to process and archive real-time ultrasonic spectra. Each ultrasonic field (sampling area) was 8 mm^2^.

Flow sensors (model 105, McMillan Co., Georgetown, TX, USA) were located on the permeate line and were connected to a 12-bit multifunction I/O analog-to-digital converter (NI-USB 6008, National Instruments, Austin, TX, USA); real-time permeate flow-rates were recorded using a laboratory PC. A multi-channel scanner was used for acquiring ultrasonic signals from the ultrasonic transducers, and a custom LabVIEW program (National Instruments, Austin, TX, USA) was used to automatically record permeate flow-rates and composite ultrasonic spectra.

After a membrane was secured in a cross-flow cell, transducers were mounted on the cell, and the pressure was adjusted to 25 psi (172 kPa). Pressure was regulated using a needle valve downstream from the cross-flow cell and monitored by pressure gauges near the cross-flow cell inlet and outlet. Both retentate and permeate were recycled to the feed tank. All experiments were conducted under laminar conditions with a cross-flow velocity of 0.01 m/s that corresponds to a Reynold's number of approximately 80 in this configuration. Cross-flow velocity was calculated using volumetric retentate measurements at the outlet of the cross-flow cell. Baseline conditions were established by running ultrapure water thorough the system for 24 h. After membrane-setting, protein solutions were selectively introduced. The BSA (Sigma-Aldrich, St. Louis, MO, USA) used had a molecular size of 67 kDa and an isolelectric point of 4.7, and the amylase utilized was from a wildtype *Bacillus subtilis α*-amylase (55 kDa) with an isoelectric point of 5.6. All feed solutions were prepared at neutral pH (7.4 ± 0.1) and an ionic strength of 0.01 M by diluting with phosphate-buffered saline. The PVDF membranes were fouled with BSA at two different challenge concentrations: 0.1 and 1 g/L. The PS membranes were fouled with BSA and with amylase, both at a challenge concentration of 0.1 g/L. During the fouling experiments with BSA, the feed temperature was kept at 24 ± 2 °C. During the fouling test with amylase, the temperature was controlled at 15 ± 2 °C with a recirculating chiller. Amylase was tested at lower temperature since this particular amylase, depending on its concentration, the pH, and other factors, has a tendency to fall out of solution above about 20 °C.

During a fouling experiment, feed was constantly mixed using a magnetic stirrer, and permeate was recycled to the feed tank. The permeate flow-rate and real-time ultrasonic spectra were continuously monitored for 5 to 25 h, depending on the fouling response. The test was terminated when the permeate flow-rate reached a steady-state value.

### 2.3. Real-Time Amplitude, Frequency and Total Reflected Power (TRP) Distributions

The reflection time (s) and amplitude (mV) of reflected sound waves were recorded and compiled into frequency distributions with a Fourier transform using commercial software (Matlab, Mathworks Inc., Natick, MA, USA) according to methods previously described [29,30]. The total reflected power (TRP) from each ultrasonic observation was determined by integrating the amplitude of reflected sound waves. TRP distributions were compiled and normalized by their TRP observations corresponding to an initial phase when the membranes were in a clean but otherwise identical operating condition [21].

The intensity of the ultrasonic energy delivered by the monitoring system used in this study is comparable to that delivered by diagnostic ultrasonic systems used for many non-destructive medical applications. Such intensity levels do not result in mechanical damage or heating of the biopolymers accumulating in or on separation membranes [21].

### 2.4. Post-Mortem Characterization

Fouled membranes were characterized by post-mortem techniques including a gravimetrically normalized biochemical assay, optical examination using scanning electron microscopy (SEM) and scanning acoustic microscopy (SAM).

#### 2.4.1. Biochemical Assay

After removing the membranes from the cross-flow cells, the region directly beneath the transducers was sectioned into coupons of known surface area (*ca*. 7 cm^2^). Water-soluble protein was then eluted from the membranes using the following procedure. Membrane coupons were aseptically placed in 50-mL clean plastic test tubes. A total of 10 mL of ultrapure sterile water was added, and the resulting solution sonicated on ice for 1 h. Eluent was analyzed for protein content using a bicinchoninic acid kit (Pierce, Rockford, IL, USA) and a BSA standard calibrator. Colorimetric results were obtained by measuring 562-nm absorbance using a spectrophotometer (model DR/2010, Hach, Loveland, CO, USA).

#### 2.4.2. Optical Examination

Concurrently with the biochemical analysis, fouled membrane coupons were prepared for high magnification optical evaluation using SEM. Cold storage (4 °C) was used to preserve membrane-associated proteins between their removal from the cross-flow cell and the SEM scan. The membrane samples were analyzed with a low-vacuum SEM (model JSM 6480LV, JOEL Ltd., Tokyo, Japan).

#### 2.4.3. Scanning Acoustic Microscopy

Membrane coupons were also characterized using an acoustic microscope operating in a scanning mode. Acoustic monitoring was non-destructively executed by traversing the membrane coupons in a constant temperature water bath using a methodology developed and described by Kujundzic and colleagues [21,22]. Independent cohorts of reflections were obtained in triplicate from the same coupon, and TRP data were taken from coupons sectioned from different parts of the membrane.

### 2.5. Statistical Evaluation

Accepted statistical analysis protocols were applied to rigorously assess differences and correlations between ultrasonic responses during membrane operations. When the PS membranes were utilized, three phases were identified: (1) membrane compaction (usually observed during the first hours of operation); (2) membrane setting (designated to be at steady state when the pure water flow-rate varied less than 4% over 12 h) [31]; and (3) acute membrane fouling (immediately following the feed challenge). When the PVDF membranes were used, compaction was not observed. In this study, the Anderson-Darling normality tests were applied to all TRP and permeate flow-rate data; this test measures the deviation of a data set from a prescribed statistical distribution, resulting in a probability (p) value. The statistical standards in this study were established by choosing a 90% confidence level. As judged by the Anderson-Darling test, some TRP spectra were not normally distributed, thereby requiring nonparametric statistical analyses. Analogous to a parametric *t*-test, a Mann-Whitney test at a 90% confidence level was employed to assess significant differences between nonparametric distributions. Acoustic spectra from the PVDF membranes fouled with BSA and acoustic spectra from the PS membranes fouled with amylase were normally distributed, requiring *t*-tests with unequal variances at a 90% confidence level to assess significant differences between TRP distributions. Acoustic spectra from the PS membranes fouled with BSA were not normally distributed, thus requiring the use of the Mann-Whitney test to assess differences between TRP distributions during compaction, setting, and fouling. In addition, permeate flow-rate data were not normally distributed. Thus, the Mann-Whitney test was used to compare permeate flow-rates during the compaction, setting, and fouling phases in all experiments.

Observations were compiled and presented in the form of box plots, where permeate flow-rate (mL/min) and TRP (mV × MHz) were isolated as process variables and averaged over the period of each operating phase. TRP values are presented for two transducers positioned on each cross-flow cell. A horizontal line in the middle of each rectangular box represents the median observation of each distribution. The minimum and maximum values are located at the endpoint of the vertical line extended through the box. The top of the box represents the 75th percentile, and the bottom of the box corresponds to the 25th percentile. Points at a greater distance from the median than 1.5 times the 75th and 25th percentile values are plotted individually as asterisks and represent potential outliers. The nonparametric, Kruskal-Wallis test was used to test for differences in the SAM TRP distributions from clean and protein fouled membranes.

## 3. Results

### 3.1. PVDF Membranes Fouled with BSA

**Figure 2:**
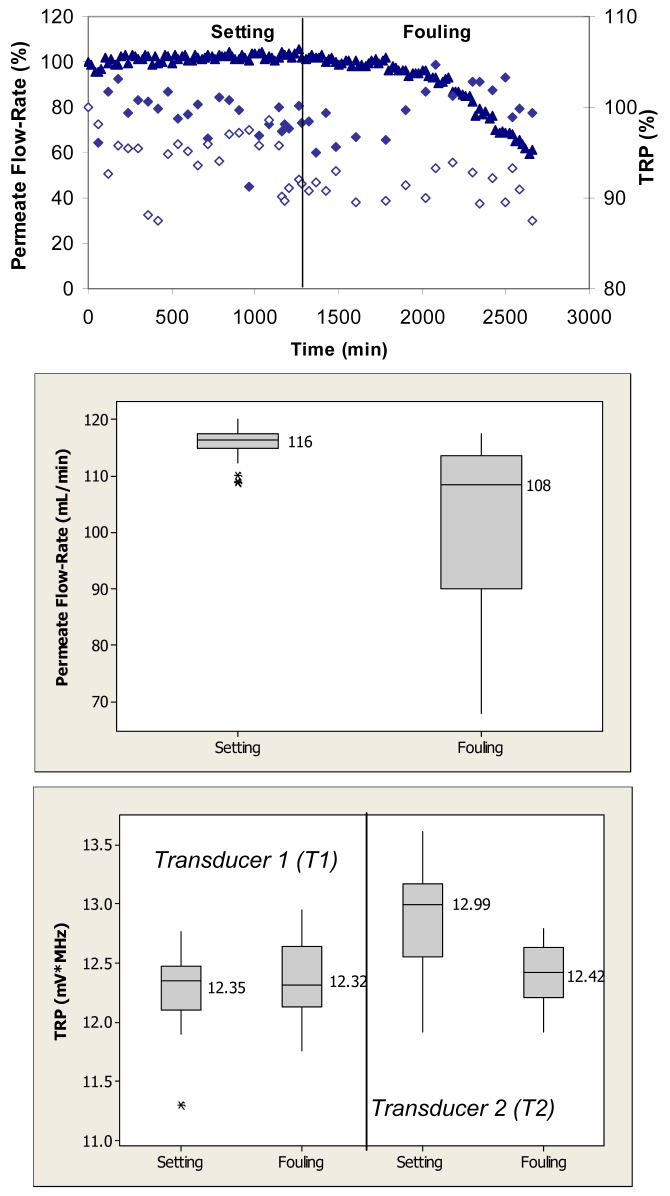
TOP: Permeate flow (▲) and total reflected power (TRP) from two transducers (◆, ◊) associated with setting and fouling when PVDF membranes were challenged with 0.1 g/L BSA; maximum standard deviations from at least three independent observations are 25% and 6% for permeate flow and TRP, respectively. MIDDLE: Permeate flow data during the challenge represented in a box plot format where the vertical lines on the boxes indicate minimum and maximum observations; upper and lower quartiles are represented by the box boundaries and the median value is represented by the central intersecting line. BOTTOM: TRP distributions from each of two transducers mounted on the flow cells during the challenge represented in a box plot format.

Figure 2 (top) shows relative changes in permeate flow and total reflected power over the two phases of cross-flow cell operation, *i.e.*, membrane setting and fouling for the case where the PVDF membranes were fouled with 0.1 g/L BSA. During the initial phase, the flow cells were operated with ultrapure water. These membranes had ultrapure water permeate flow-rates starting at 120 mL/min. Over the first 1250 min, the permeate flow-rate remained relatively constant. Following the introduction of BSA, the permeate flow-rate decreased by more than 40% over the subsequent 1500-min period. The total reflected power values changed between 5% and 10% during the fouling challenge.

Permeate flow-rate data were averaged over the entire time period of the setting and fouling phases and are presented in box plots for each phase. When applied to the permeate flow-rate data, a nonparametric Mann-Whitney test showed that the flow-rates during setting were significantly different from the flow-rates during the fouling phase (p < 0.1) (Figure 2, middle). TRP values presented in the box plots were also averaged over the entire time period for each operating phase. When applied to the TRP distribution spectra obtained during membrane challenges with 0.1 g/L BSA, *t-*tests showed that the TRP response during setting was not significantly different from the TRP response during the fouling phase on one transducer (p > 0.1) and significantly different on the other transducer (p < 0.1) (Figure 2, bottom).

**Figure 3:**
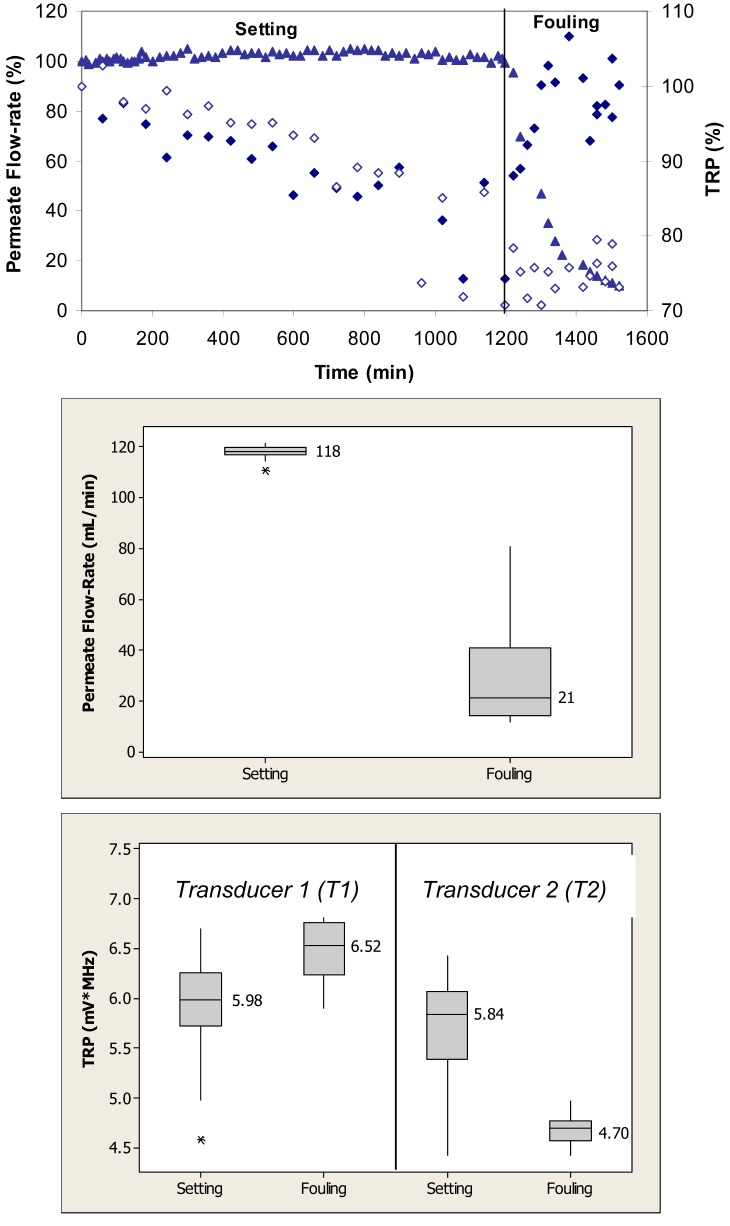
TOP: Permeate flow ((▲) and total reflected power (TRP) from two transducers (◆, ◊) associated with setting and fouling when PVDF membranes were challenged with 1 g/L BSA; maximum standard deviations from at least three independent observations are 6% and 22% for permeate flow and TRP, respectively. MIDDLE: Permeate flow data during the challenge represented in a box plot format where the vertical lines on the boxes indicate minimum and maximum observations; upper and lower quartiles are represented by the box boundaries and the median value is represented by the central intersecting line. BOTTOM: TRP distributions from each of two transducers mounted on the flow cells during the challenge represented in a box plot format.

Figure 3 (top) shows relative changes in permeate flow and total reflected power over the two phases of cross-flow cell operation, *i.e.*, membrane setting and fouling for the case where the PVDF membranes were fouled with 1 g/L BSA. During the initial phase, the flow cells were operated with ultrapure water. Over the first 1200 min, the permeate flow-rate remained relatively constant. Following the introduction of BSA, the permeate flow-rate decreased by 90% over the subsequent 300-min period. The total reflected power values changed by as much as 30% during the fouling challenge.

When applied to the permeate flow-rate data, a nonparametric Mann-Whitney test showed that the flow-rates during setting were significantly different from the flow-rates during the fouling phase (p < 0.1) (Figure 3, middle). When applied to the TRP distribution spectra obtained during membrane challenges with 1 g/L BSA, a *t-*test showed that the TRP response during setting was significantly different from the TRP response during fouling phase for both transducers (p < 0.1) (Figure 3, bottom).

### 3.2. Polysulfone Membrane Fouled with BSA and Amylase

**Figure 4:**
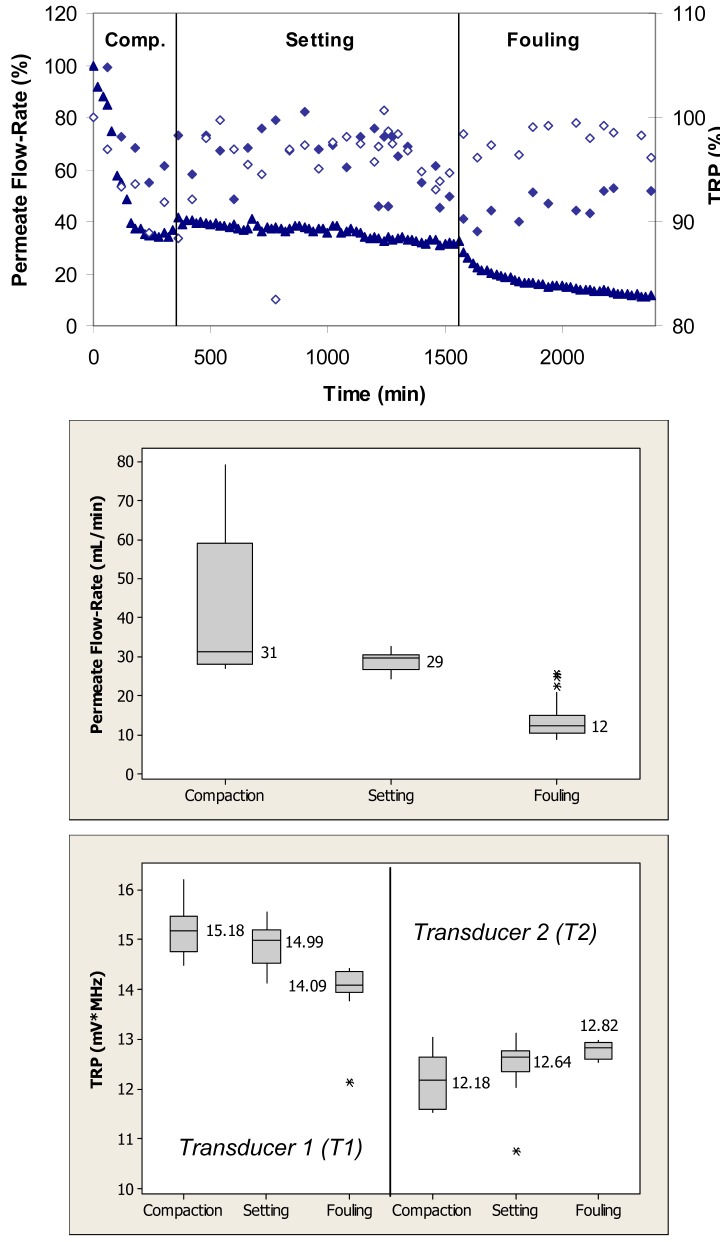
TOP: Permeate flow (▲) and total reflected power (TRP) from two transducers (◆,◊) associated with compaction, setting and fouling when PS membranes were challenged with 0.1 g/L BSA; maximum standard deviations from at least three independent observations are 6% and 7% for permeate flow and TRP, respectively. MIDDLE: Permeate flow data during the challenge represented in a box plot format where the vertical lines on the boxes indicate minimum and maximum observations; upper and lower quartiles are represented by the box boundaries and the median value is represented by the central intersecting line. BOTTOM: TRP distributions from each of two transducers mounted on the flow cells during the challenge represented in a box plot format.

Figure 4 (top) shows relative changes in the permeate flow and total reflected power over the three phases of flow cell operation, *i.e.*, membrane compaction, setting, and fouling for the case where the PS membrane was fouled with 0.1 g/L BSA. During the initial phase, the flow cells were operated with ultrapure water. This membrane had initial ultrapure water permeate flow-rate values between 50–60 mL/min, while the fully compacted ultrapure water flow-rate was approximately 30 mL/min. Over the first 300 min, the permeate flow-rate decreased by approximately 60% due primarily to the structural aspects of membrane compaction. Subsequently, permeate flow-rate remained relatively constant over next 1200 min. Following the introduction of BSA, permeate flow-rate decreased from a value equivalent to 35% to one equivalent to 15% of the initial maximum value over the subsequent 840-min period. The total reflected power values decreased <10% during the fouling challenge.

When applied to the permeate flow-rate data, a nonparametric Mann-Whitney test showed that the permeate flow-rates during compaction and setting were significantly different from the flow-rates during the fouling phase (p < 0.1) (Figure 4, middle). When applied to the TRP distribution spectra obtained during membrane challenges with 0.1 g/L BSA, a Mann-Whitney test indicated that the TRP response during the compaction phase was not significantly different from the TRP response during the setting phase on one transducer (p > 0.1), but significantly different on the other transducer (p < 0.1) (Figure 4, bottom). The TRP response during the compaction and setting phases was statistically different from the TRP response during the fouling phase on both transducers (p < 0.1). Inconsistency in absolute TRP values obtained from transducers T1 and T2 from the ultrapure water phase is most likely due to local differences in the membrane itself including variability in the membrane thickness at the two transducer locations.

**Figure 5:**
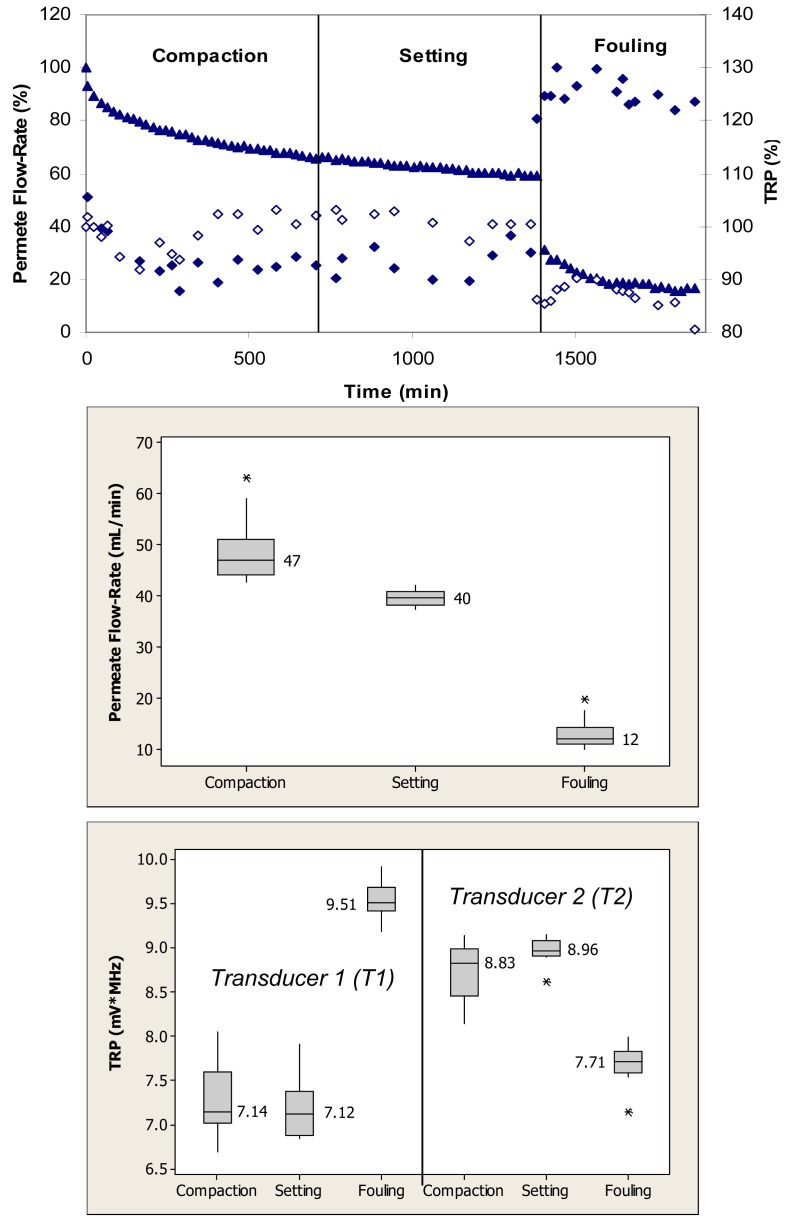
TOP: Permeate flow (▲) and total reflected power (TRP) from two transducers (◆,◊) associated with compaction, setting and fouling when PS membranes were challenged with 0.1 g/L amylase; maximum standard deviations from at least three independent observations are 5% and 19% for permeate flow and TRP, respectively. MIDDLE: Permeate flow data during the challenge represented in a box plot format where the vertical lines on the boxes indicate minimum and maximum observations; upper and lower quartiles are represented by the box boundaries and the median value is represented by the central intersecting line. BOTTOM: TRP distributions from each of two transducers mounted on the flow cells during the challenge represented in a box plot format.

Figure 5 (top) shows relative changes in permeate flow and total reflected power over the three phases of flow cell operation, *i.e.*, membrane compaction, setting, and fouling for the case where the PS membranes were fouled with 0.1 g/L amylase. During the initial phase, the flow cells were operated with ultrapure water. Over the first 720 min, the permeate flow-rate decreased by almost 40% due primarily to the structural aspects of membrane compaction. Subsequently, the permeate flow-rate remained relatively constant over the approximately 720-min setting phase. Following the introduction of amylase, the permeate flow-rate decreased from a value equivalent to 60% to one equivalent to 20% of the initial maximum value over the subsequent 480-min period. The total reflected power values changed by as much as 30% during the fouling challenge.

When applied to the permeate flow-rate data, a nonparametric Mann-Whitney test showed that the flow-rates during compaction and setting were significantly different from the flow-rates during the fouling phase (p < 0.1) (Figure 5, middle). When applied to the TRP distribution spectra obtained during the membrane challenges with 0.1 g/L amylase, a *t-*test showed that the TRP response during the compaction phase was not significantly different from the TRP response during the setting phase for one transducer (p > 0.1), and different for the other transducer (p < 0.1) (Figure 5, bottom). The TRP response during the compaction and setting phases was significantly different from the TRP response during the fouling phase for both transducers (p < 0.1).

### 3.3. Post-Mortem Characterization Results

#### 3.3.1. Biochemical Assay

Protein associations with the MF membranes varied depending on the challenge protein, fouling time, type of membrane and protein concentration. The protein concentration associated with the PVDF membranes after 1500 min of fouling with 0.1 g/L BSA was 34 ± 17 μg/cm^2^. For comparison, the protein concentration after 300 min of fouling with 1 g/L BSA was 81 ± 9 μg/cm^2^. When the PS membranes were fouled with BSA at a concentration of 0.1 g/L, the protein concentration associated with the membranes after 840 min of fouling was 39 ± 2 μg/cm^2^. When the PS membranes were fouled with amylase at a concentration of 0.1 g/L, the protein concentration after 480 min of fouling was 14 ± 11 μg/cm^2^.

#### 3.3.2. Scanning Acoustic Microscopy

**Figure 6:**
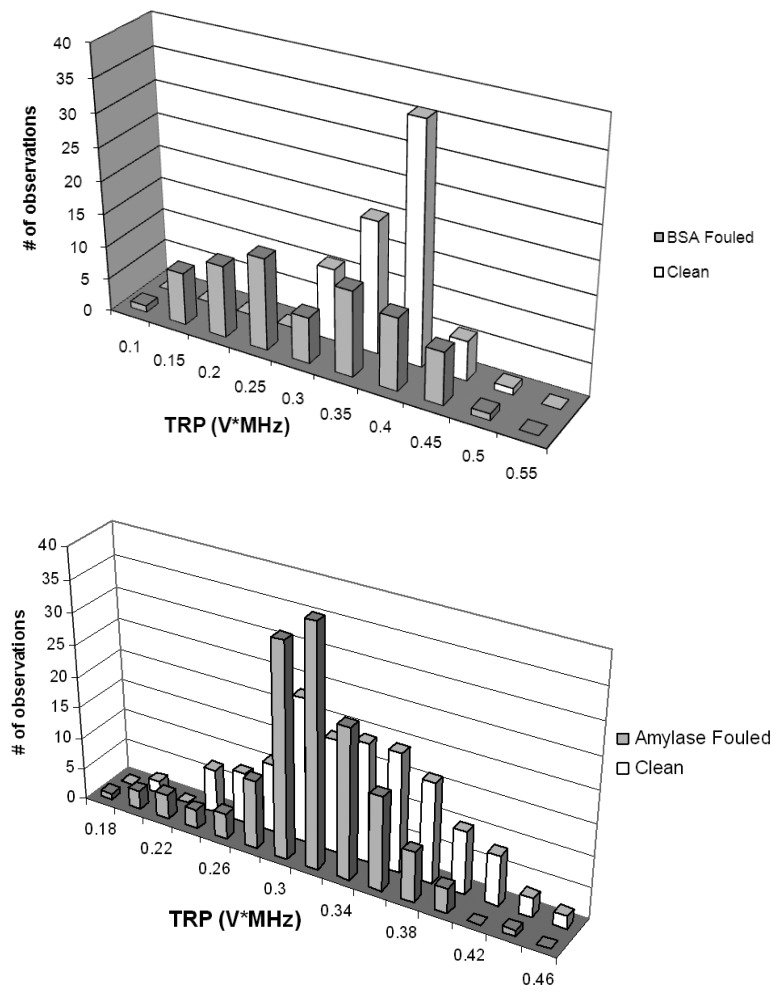
Total reflected power (TRP) distributions obtained via scanning acoustic microscopy (SAM); mean and standard deviation of the distributions in units of VxMHz are presented in parentheses; TOP: a virgin PVDF membrane coupon (0.37 ± 0.06) and a BSA-fouled PVDF membrane coupon (0.32 ± 0.11); BOTTOM: a virgin PS membrane coupon (0.34 ± 0.06) and an amylase-fouled PS membrane coupon (0.35 ± 0.05).

Acoustic spectra from virgin PVDF and PS membrane materials (prepared with ultrapure water) and from coupons sectioned from membranes fouled with BSA and with amylase are shown in Figure 6. The fouling mechanism associated with a relatively pure protein feed solution would be expected to be dominated by internal mechanisms under the test conditions here, although SEM results described later showed some surface accumulation of protein. The findings indicate that UR could differentiate between clean and fouled membranes with a relatively high degree of confidence (90%) for the challenge with replication (*n* = 3). PVDF membrane coupons fouled with BSA showed a bimodal and somewhat wider TRP distribution when compared to virgin membrane coupons. As judged by nonparametric Kruskal-Wallis test, TRP distributions from clean and BSA-fouled membranes were significantly different (p < 0.1). The membrane coupons fouled with amylase showed a slight TRP departure toward higher power values when compared to virgin membrane coupons; a nonparametric test indicated that the two TRP distributions obtained from virgin and amylase-fouled coupons were also significantly different (p < 0.1).

#### 3.3.3. Optical Examination

**Figure 7:**
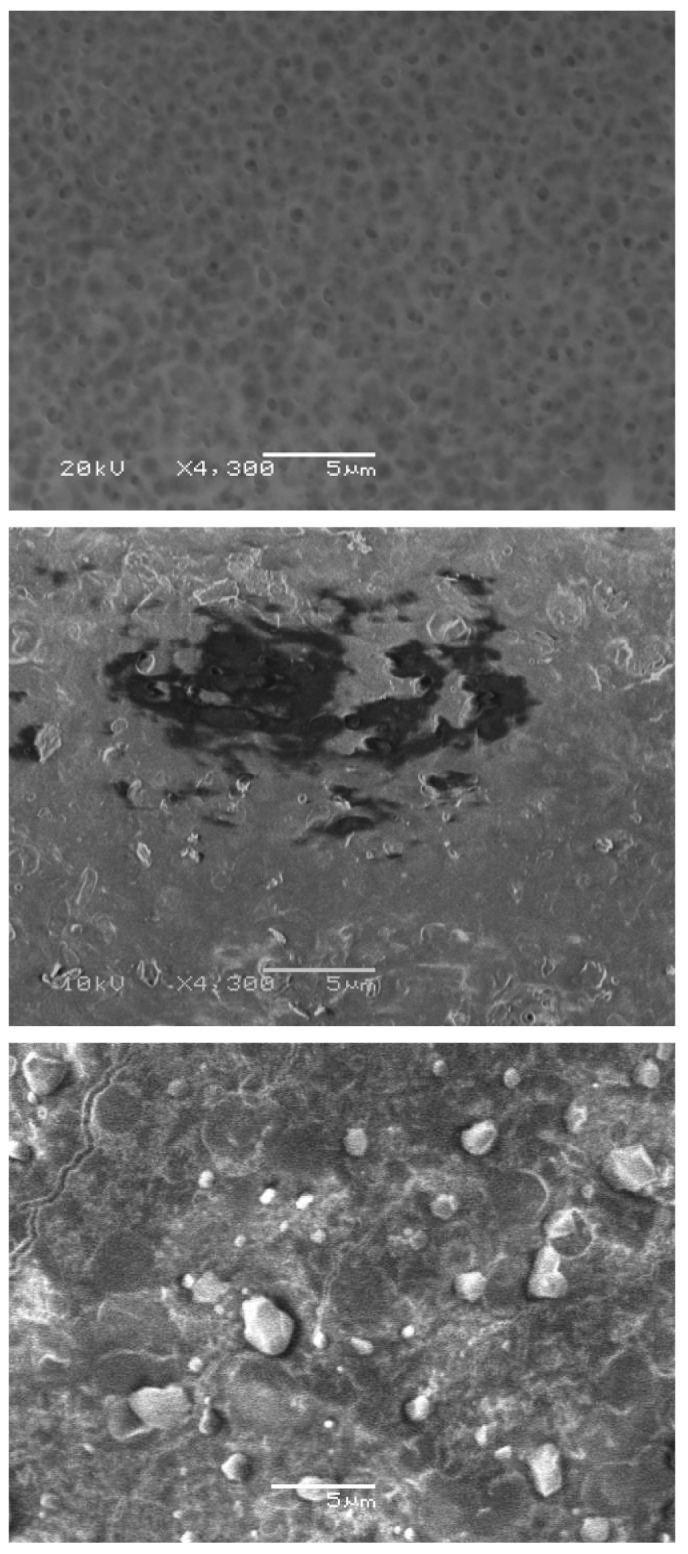
Representative SEM micrographs of polysulfone membranes. TOP: clean with the porous surface clearly visible; MIDDLE: fouled with 0.1 g/L BSA; BOTTOM: fouled with 0.1 g/L amylase. Original magnification: 4300×.

Optical inspection via SEM complemented the results of the real-time acoustic scans and suggested that a discontinuous accrual of membrane-associated biopolymers was the dominant mode of biomass accumulation on the membrane surfaces. Representative SEM images of a virgin PS membrane sample (Figure 7, top) as well as samples from a PS membrane fouled with 0.1 g/L BSA (Figure 7, middle) and a PS membrane fouled with 0.1 g/L amylase (Figure 7, bottom) confirmed the “patchy” distribution of biomass on the surfaces of the membranes fouled with both proteins.

## 4. Discussion

By systematically observing the responses of two different MF membranes challenged with different proteins at different concentrations, we obtained converging lines of acoustic, optical, and gravimetric evidence which suggests that at protein concentrations as low as 0.1 g/L, UR (accompanied with other post-mortem and real-time observations) was able to monitor protein fouling in real time. The decline in permeate flow-rate observed with the PS membranes during operation with ultrapure water is most likely due to membrane compaction [32]. Similarly, in the study by Li and colleagues [33] with nylon membranes (nominal pore size of 0.2 μm) operating with pure water for 2 h at a pressure of 150 kPa, observed significant decrease (76% maximum) in permeate flow-rate was also attributed to structural compaction. Compaction was not observed in our tests using PVDF membranes. As indicated previously, these membranes are much thinner than the PS membranes and do not contain a support structure, which is highly porous and undoubtedly prone to significant compaction.

The accuracy of the transducers was assessed using a simple and standard protocol. The transducers were mounted atop an aluminum reference block of known thickness (26.6 mm) and ultrasonic data were then sequentially obtained from each transducer. Based on the arrival times corresponding to the ultrasonic reflections from the top and bottom of the reference block and the wave velocity in aluminum (6420 m/s), the thickness of the block was measured. Representative results from two transducers were identical (26.64 mm) and were statistically identical to a thickness value of 26.58 mm determined independently using a calibrated micrometer.

In earlier studies we have experimented with multiple transducers in different locations above a cross-flow cell. Since our approach considers measuring TRP changes in real-time, transducers observed reflection changes at pre-selected (sentinel) locations, and were not moved as they recovered reflective power inventories. In this study, we selected to place transducers at the cell intake where highest amount of organic fouling is expected to be seen. Variability in the absolute TRP values from transducers T1 and T2 during the ultrapure water phase with the PS membrane (Figure 4 and Figure 5, bottom) can be explained by local differences in the membrane including thickness at the two transducer locations. Studies of local differences have been reported in the literature. For example, in a test of material homogeneity, Evans [34] tested three types of polymeric membrane coupons randomly cut from different areas of the same membrane roll. Results suggested that the ultrasonic TRP baseline for membrane coupons from the same manufacturing lot significantly differ and that considerable material variations exist within commercial membrane rolls. In addition, different local behavior of PS membrane during compaction and setting (Figure 4 and Figure 5 top) can be explained by membrane sample-to-sample variations in the same manufacturing lot [34,35].

When membranes were fouled in the cross-flow cell module, the degree of TRP departure varied greatly and was not always consistent among co-located transducers. This may be explained by non-uniform distribution of the organic fouling layer over the membrane surface, *i.e.*, “patchiness” which was evident from SEM images (Figure 7, middle and bottom). Whereas permeate flow-rate data are represented by a response that is effectively averaged over the entire membrane surface, UR provides a near-point measurement that characterizes the local condition. These differences were determined to be statistically significant and are not unexpected given that the organic fouling layers are dynamic and viscoelastic in nature, *i.e.*, the amorphous protein deposits possess hydrogel-like characteristics such that the deposits can move along or sporadically attach or detach from the membrane surface [21,22,23]. The importance of such local differences relative to the flux behavior is essential with respect to the utility of UR. In the present case for which the membrane axial dimension is small, fouling and permeate responses occur on the same time scale so UR offers relatively little practical advantage. In situations in which the membrane axial dimension is sufficiently large such that organic fouling develops preferentially near one location, UR would be expected to show a statistically significant departure from baseline values before a corresponding decrease in permeate flow [23]. This possibility of detecting early-stage fouling could be advantageous with respect to optimizing the cleaning of an organic foulant as has been reported for inorganic scaling [15,18], or even in improving membrane module design [36].

When the PVDF membranes were fouled with BSA in the cross-flow cells, a non-systematic change in TRP values was observed. These results are different than those obtained for the same polymeric membrane but with a larger nominal pore size (0.65 μm) that was fouled with bacterial biofilm and analyzed post-mortem via scanning acoustic microscopy [21]. In that study, TRP distributions showed an initial increase followed by a significant decrease in TRP values. This observed difference in TRP values could be related to the different modes associated with fouling via bacterial biofilm formation versus fouling with a specific biopolymer (*i.e.*, protein). Also, these responses can be explained by differences in the relative acoustic impedance of the various layers as well as increased sensitivity to internal fouling (bulk density changes) versus surface-associated fouling. Membranes with larger pore sizes tend to experience both surface and internal fouling, whereas membranes with a smaller pore size experience primarily surface fouling. Moreover, real-time changes in the fouling layers are more difficult to document than the time independent responses characterized by the post-mortem techniques. Significant variability in the TRP values was evident during the course of our tests and may be attributed to the fact that these organic foulants manifest as hydrogels, which are dynamic in nature with a morphology that changes due to substrate concentrations and local hydrodynamic conditions. In addition, a portion of TRP variability from fouling may be attributed to capricious layer growth, movement and sloughing.

In cross-flow experiments reported here, the concentration of the protein on the membrane surface at the end of the fouling phase was 14–81 μg/cm^2^, thus demonstrating that UR can be sensitive to very low protein concentrations. In a related study [22], 0.2 μm PVDF membranes were fouled with BSA in flow cells operating in a dead-end mode at a transmembrane pressure of 13.8 kPa (2 psi). BSA solution with a concentration of 1 g/L was prepared and then filtered through the MF membranes, and coupon sections were obtained for acoustic analysis in scanning mode. When compared to clean membranes used as a reference, BSA-fouled membrane coupons evidenced consistent attenuations in reflection amplitude, which caused statistically significant departures in reflected power. The findings based on the ultrasonic response of the protein-fouled membranes showed that UR can differentiate between clean and protein-fouled membranes.

When protein fouling on the PVDF membranes tested at two different BSA concentrations (0.1 and 1 g/L) was compared, more intense fouling was achieved at shorter time intervals when the membranes were challenged with higher BSA concentration. In addition, differences in the TRP responses between the setting and fouling phases were more pronounced when this membrane material was fouled with higher BSA concentration. Comparison of the fouling potential of the PVDF and PS membranes using the same protein at the same challenge concentration indicated that fouling occurred more quickly with the latter. In addition, the trends for the permeate flow-rate decreases were different. Whereas the PS membrane evidenced an immediate decrease in the permeate flow-rate, the decline for the PVDF membrane was initially less evident in the early stage but was more pronounced after 600 min of fouling. In addition, changes in the TRP responses were greater for the PS as compared to the PVDF membranes. For the case in which the PS membranes were fouled with two different proteins, BSA and amylase, at the same concentration, the fouling response was detected sooner with amylase. In addition, a more significant change in the TRP response was observed with amylase when compared to BSA.

Post-mortem characterization data agreed well with permeate flow-rate behavior and real-time ultrasonic spectra. In the test where PVDF membranes were fouled for 1500 min with 0.1 g/L BSA, the decrease in permeate flow-rate was 40%, which corresponded to a protein concentration of approximately 34 μg/cm^2^. An almost 2.5-times higher protein concentration was measured on the same membrane that was fouled (for only 300 min) with an order of magnitude higher BSA concentration that resulted in 90% decrease in permeate flow-rate. Similar protein concentrations were obtained when both membranes were fouled with 0.1 g/L BSA, which resulted in a similar TRP response. As expected, post-mortem characterization via SAM showed that ultrasonic spectra from clean and fouled membrane coupons were statistically different at a confidence level of 90%.

Overall, our results indicated that UR could be successfully used to detect protein fouling associated with different commercial polymeric MF membranes. Both real-time and post-mortem characterization techniques showed that UR could be successfully used to monitor protein fouling of MF membranes. Despite the success of the UR approach in monitoring protein fouling, it is clear that more specific information regarding the onset, chemical nature and thickness of protein layers would be of great value in optimizing module operation including the evaluation of particular cleaning strategies and optimization of the technique in industrial-scale applications.

## 4. Conclusions

This study describes the application of UFDR for a non-invasive method to detect and monitor protein fouling on commercial polymeric MF membranes. The ultrasonic signal response corresponded well with permeate flow-rate data. UFDR was able to detect the onset of protein fouling and monitor protein fouling in the cross-flow modules. Although, the degree of TRP departure varied significantly in a manner that was not always predictable, statistical analysis demonstrated that the TRP response changed significantly as a result of membrane fouling with proteins which are commonly used to model organic fouling processes. Our findings provide a strong basis for continued development of UFDR methodology for monitoring protein fouling in MF separations. Such efforts must incorporate adaptations for successful transition from bench- to large-scale industrial applications.
